# Plant3R: Fusing 3D feature learning with Gaussian splatting to enhance wheat plant 3D reconstruction precision

**DOI:** 10.1016/j.plaphe.2026.100200

**Published:** 2026-03-21

**Authors:** Jiateng Ma, Xiaolong Hu, Liangsheng Shi, Yufan Zhang, Yixiang Jiang, Hao Zhang, Shuo Duan

**Affiliations:** a State Key Laboratory of Water Resources Engineering and Management, Wuhan University, Wuhan, China; b School of Artificial Intelligence, Wuhan University, Wuhan, China

**Keywords:** Plant 3D reconstruction, Wheat phenotyping, MASt3R, 3D Gaussian splatting

## Abstract

Precise reconstruction of plant phenotypes is crucial for smart agriculture. Conventional methods struggle with low efficiency and strong dependency on high-quality data, especially for low-texture and structurally complex crops like wheat. We propose a novel 3D reconstruction framework—Plant3R—that fuses deep feature learning with 3D Gaussian Splatting (3DGS). It innovatively uses the Matching and Stereo 3D Reconstruction (MASt3R) model for sparse point cloud reconstruction and camera pose estimation via its 3D feature matching capabilities, which substantially improve image matching rates and the quality of sparse point clouds. Subsequently, 3DGS is employed for rendering and optimization, enabling end-to-end, high-fidelity, and high-robust 3D reconstruction of wheat plants. Validated on potted wheat at multiple growth stages using handheld images, our experimental results demonstrate that Plant3R performs well in feature extraction and matching, and the reconstructed point cloud provides a good geometric prior for the subsequent rendering stage. In most scenes, its key rendering metrics—Peak Signal-to-Noise Ratio (PSNR) > 34, Structural Similarity Index Measure (SSIM) of 0.94, and Learned Perceptual Image Patch Similarity (LPIPS) < 0.26—surpassed Neural Radiance Fields (NeRF) and the original 3DGS. Moreover, extracted phenotypic traits such as plant height, leaf length, and width showed high correlation with manual measurements (R^2^ > 0.94), confirming its utility for accurate and quantitative phenotype analysis. Overall, Plant3R not only improves the rendering quality and geometric precision of 3D modeling, but also provides a reliable tool for accurate phenotypic parameter extraction and high-throughput crop phenotyping in precision agriculture.

## 1 Introduction

Plant phenotyping serves as a critical bridge between genotypes and agronomic performance, providing the basis for understanding how plants respond to genetic and environmental factors [1]. Against the backdrop of global food security and climate change, accurate and scalable phenotyping has become essential for breeding resilient crop varieties and guiding precision agriculture [2,3]. Meanwhile, the rapid development of consumer-grade sensing technologies and artificial intelligence is significantly accelerating the modernization of smart agriculture, leading to a notable increase in agricultural production efficiency [4]. In this process, the rapid and accurate acquisition of crop phenotypic information is considered a powerful tool for dynamic monitoring in crop breeding, and a foundation for intelligent agricultural management [5]. Traditional phenotyping methods typically rely on manual measurements, which are labor-intensive, inefficient, and somewhat destructive, while also being susceptible to subjective influences [6,7]. Although 2D image-based phenotyping methods mitigate these issues to some extent, they are limited by spatiotemporal dimensions, making it difficult to accurately characterize the complex 3D morphological changes during crop growth [8]. In contrast, crop 3D reconstruction technology non-invasively acquires the plant's volumetric structural information [9]. This not only allows for a complete record of key phenotypic features such as plant architecture and organ spatial topological relationships [10]—providing a data foundation for Functional Structural Plant Models (FSPM) [11], but also enables the revelation of the dynamic response of crop phenotypic plasticity to environmental stress through 3D data analysis [12]. This capability offers crucial guidance for crop variety improvement and optimizing cultivation management strategies.

In recent years, 3D reconstruction technology has been widely applied in crop phenotyping research [13]. Active 3D reconstruction techniques, such as TOF cameras [14,15], Lidar [16,17] and laser scanners [18], are highly favored for their ability to provide high-precision 3D models. For example, Vázquez-Arellano et al. used a TOF camera to reconstruct 3D models of maize and extracted plant height information, controlling the average error to within 8.7 mm [19]; Zheng et al. utilized a 3D digitizer to scan wheat plants for digital visualization, extracting phenotypic parameters such as leaf length, leaf width, and leaf inclination angle, with corresponding coefficients of determination (R^2^) reaching 0.93, 0.98, and 0.85 [20]; Nadeem et al. used UAV LiDAR to obtain multi-temporal point cloud data of crop canopies and validated the effect of different flight paths on extracting canopy heights of various crops [21]. However, these methods generally face challenges of high equipment cost and complex operation, often requiring multiple calibrations and repeated scanning to complete point cloud matching and stitching, which severely limits their widespread adoption in practice [22].

To overcome these limitations, many researchers have turned to passive imaging methods, relying on passive sensors like cameras to generate 3D crop models by processing images from different viewpoints. Among numerous techniques, the SfM-MVS algorithm [23] has received significant attention for plant model reconstruction due to its advantages such as low hardware cost and high color fidelity. It estimates camera parameters and 3D point clouds through feature matching across multiple overlapping images. However, its reconstruction process has high requirements for image quality and viewpoint coverage, involves considerable computational complexity, and requires a long processing time. This is especially prone to errors and reconstruction failures when dealing with complex crop structures like overlapping wheat leaves. Thus, researchers began integrating deep learning into 3D reconstruction networks. For instance, Liu et al. proposed ReC-MVSNet, which integrates a reparameterization structure into a point cloud 3D reconstruction network to enhance the model's complex feature extraction capabilities, improving its accuracy by nearly 43.3% [24]. Similarly, He et al. combined GRNN with SfM-MVS to extract trait parameters of soybean plants, achieving a MAPE as low as 2.7% for plant height extraction [25]. Additionally, Wang et al. utilized thermal infrared images and stereo vision to acquire 3D data of potatoes with temperature information, used for analyzing the 3D distribution of the Crop Water Stress Index (CWSI) [26]. While these methods improved performance by optimizing networks, they did not fundamentally change the underlying principles of reconstruction.

The proposal of Neural Radiance Fields (NeRF) [27] in 2020 marked a revolutionary breakthrough. It has achieved high-quality reconstruction through deep learning and demonstrated immense application potential in botany. Hu et al. were the first to introduce NeRF into crop phenotyping analysis, achieving high-fidelity reconstruction of various crop scenes and releasing a related dataset, which laid the foundation for subsequent research [28]. Yang et al. developed PanicleNeRF to address the phenotyping of rice panicles, successfully extracting panicle inflorescence traits by combining it with the SAM and YOLOv8 models, with an R^2^ of up to 0.8 compared to ground truth values [29]. However, the implicit representation of NeRF still poses a major challenge for quantitative analysis: it is difficult to directly extract and quantify phenotypic parameters from the model, complicating cross-scene analysis. The emergence of 3DGS [30] has perfectly addressed this problem. Compared to NeRF's implicit representation, 3DGS achieves a significant breakthrough in rendering efficiency through explicit Gaussian distribution optimization; its training results can be saved and edited, providing a natural data foundation for the extraction and calculation of phenotypic parameters. Currently, 3DGS has been applied to crop 3D phenotyping extraction and analysis with great success [31]. For example, Shen et al. proposed a foreground-segmentation-based 3D Gaussian splatting method-PlantGaussian, to achieve high-fidelity plant reconstruction across space, time, and scenes [32]; Zhang et al. integrated 3DGS with the SAM model to achieve precise 3D reconstruction and measurement of wheat spikes under field conditions [33]; furthermore, Song et al. combined 3DGS with an improved YOLOv8 model, proposing the concept of “digital cousins” to boost the model's detection performance to over 90.7% [34]. Despite these significant advancements, existing technologies still face the following challenges in meeting the demands of crop phenotyping research for fast, accurate, and low-cost 3D reconstruction: I) Traditional 3D reconstruction methods often struggle with incomplete or distorted geometry and cumulative noise, especially in regions with complex or low-texture structures. II) Recent approaches like NeRF and 3DGS, which rely on SfM preprocessing, are highly sensitive to input quality, making them prone to failure when data is incomplete or captured under unconstrained conditions.

To address these challenges, we propose Plant3R, an unconstrained highly robust reconstruction pipeline that integrates the deep feature learning of MASt3R [35] with high-fidelity rendering of 3DGS. Plant3R leverages MASt3R's robust feature matching and stereo reconstruction capabilities to provide reliable initialization, while adaptive Gaussian adjustment enables the efficient optimization of the complex anisotropic structures of wheat. This design ensures both computational feasibility and reconstruction fidelity across different growth stages. To our knowledge, this is the first application of a combined MASt3R and 3DGS model in the field of crop reconstruction. The main contributions of this study are as follows: 1) We propose a novel 3D reconstruction framework, Plant3R, which, for the first time, fuses deep feature learning from the MASt3R model with the efficient differentiable rendering of 3DGS. 2) The hybrid paradigm leverages adaptive Gaussian primitive adjustment to simulate the anisotropic 3D structure of wheat, achieving high-fidelity reconstruction while ensuring computational feasibility. 3) The framework enables efficient high-fidelity reconstruction from a small number of unconstrained images, significantly reducing computational cost and improving the practicality of 3DGS-based plant modeling.

## 2 Materials and methods

### 2.1 The pipeline of Plant3R

**Fig. 1:**
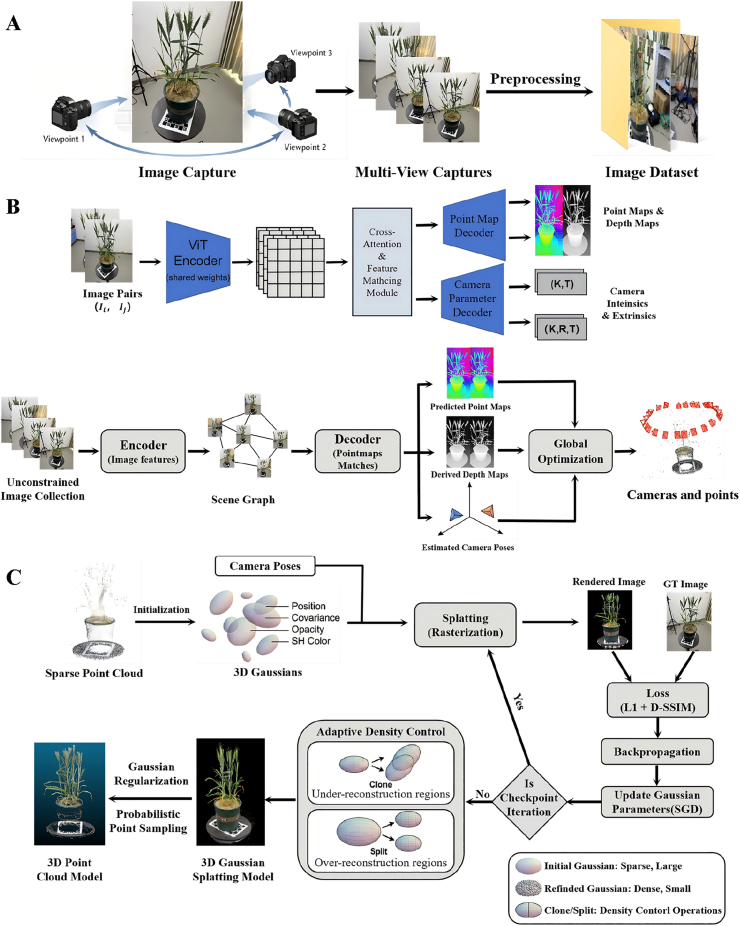
Overview of the Plant3R model's pipeline.

This study proposes the Plant3R algorithm, which fuses 3D feature learning with the 3D Gaussian Splatting model, selecting potted wheat as the research object to reconstruct 3D model of wheat plants across multiple growth stages. As illustrated in Fig. 1, the entire 3D reconstruction pipeline of Plant3R primarily consists of three steps: I) wheat image acquisition and data preprocessing; II) sparse reconstruction and camera pose estimation; Ⅲ) high-fidelity plant rendering and high-quality geometric model extraction. In the preprocessing stage, to ensure the algorithm's universality for datasets with inconsistent viewpoints or missing metadata, this study innovatively applies initial scene construction and camera pose estimation based on 3D point map regression to the wheat reconstruction process, outputting SfM format data to provide high-quality geometric priors for subsequent rendering. Subsequently, the sparse point cloud and camera poses are input into the 3DGS model, combined with adaptive density control and a tile-based rasterizer to achieve high-fidelity 3D plant rendering. For convenient extraction and measurement of 3D phenotypic parameters, the 3D Gaussian Splatting scene undergoes additional optimization, and finally, poisson surface reconstruction is employed to generate a high-quality geometric model.

### 2.2 Image data acquisition

The experimental subject selected for this study was wheat, and potted plant experiments were conducted at the Irrigation and Drainage Experimental Field of Wuhan University (30.54°N, 114.36°E). Wheat plants were cultivated in pots with a diameter of 24 cm and a depth of 24 cm, filled with 9 kg of air-dried soil. We used a standard pot cultivation method, with 5-10 seeds sown per pot. After seedlings reached the three-leaf stage, thinning was performed, retaining two to three seedlings with similar growth vigor per pot. Supplementary Fig. S1 shows the schematic diagram of the initial placement of potted plants in the experimental field. Ample water and nutrients were provided throughout the entire growth cycle of the wheat. Potted plants with significant morphological differences at various growth stages, including tillering, jointing, grain filling, and maturity, were used for data acquisition. At each growth stage, twelve pots of sample data were collected. For each pot, 30 RGB images with approximately 75-80% overlap between adjacent views were captured around the plant (covering approximately 360°) using an iPhone14 mobile device with a resolution of 4032 x 3024 pixels, a 1.0x (26 mm equivalent) focal length, and an aperture of f/1.5. Detailed camera parameters and acquisition settings are provided in Supplementary Table S1. To demonstrate the stability of the Plant3R model proposed in this study when processing sparse data or datasets lacking metadata, image data were collected in an unconstrained manner. The robustness and universality of the model were validated by reconstructing 3D point clouds from images of wheat potted plants collected at key growth stages.

### 2.3 Sparse point cloud and camera pose estimation via MASt3R

The preprocessing stage of image data, which involves estimating sparse point clouds and camera parameters, provides the data foundation for the dense reconstruction and high-fidelity rendering of the 3D Gaussian Splatting model, significantly impacting the accuracy and precision of the reconstruction outcomes. However, the sparse reconstruction process in traditional SfM algorithms is segmented into multiple subtasks, where reconstruction errors accumulate throughout the pipeline, often resulting in reconstruction failure. To overcome these limitations, we chose to fuse the matching module with the MASt3R model with the ASMK retrieval pipeline. Unlike conventional methods that approach image matching as a 2D problem, we treat it as a 3D task, leveraging point map regression to achieve initial scene reconstruction and global camera alignment.

(1) Sparse reconstruction and camera pose estimation

First, we perform image matching on the input images and complete local pairwise reconstruction. An effective and scalable MASt3R encoder integrated with Aggregated Selective Match Kernels (ASMK) [36]are employed to achieve efficient image retrieval and generate a similarity matrix $S\epsilon {\left[0,1\right]}^{N\times N}$. To obtain a small number of pairs, we select a fixed number $N_{a}=20$ of key images using farthest point sampling (FPS), and the remaining images are connected to their nearest keyframe as well as their k (k = 10) nearest neighbors, forming a visibility graph G for subsequent optimization, where edges e=(n,m) link potentially overlapping image pairs $\left(I^{n},I^{m}\right)$.For each edge e=(n,m)ϵE in the graph, a lightweight ViT network is utilized to conduct bidirectional feature matching and employ the union operation of $f\left(I^{n},I^{m}\right)\;\text{and}\;f\left(I^{m},I^{n}\right)$ operations to eliminate dependency on the order of input images. Based on the geometric features from the encoder, four point map types $:X_{n,n}$, $X_{n,m}$, $X_{m,n}$, $X_{m,m}$ are generated through implicit neural field regression, where $X_{n,m}\epsilon R^{H\times W\times 3}$ represents a 2D-to-3D mapping [35] from image $I^{n}$ to 3D points in the coordinate system of image $I^{m}$, maintaining robustness to photometric and geometric variations. Sparse correspondences are then extracted using Fast Nearest Neighbor (FastNN) search [35].

A weighted-average-based canonical point map generation mechanism is designed to mitigate single-edge estimation uncertainty: For an image $I^{n}$, its connected edge set ${ℇ}^{n}=\{e|e\;\epsilon \;E\;⋀n\;\epsilon \;e\}$ is defined as all image pairs sharing scene overlap with $I^{n}$. Noise suppression is achieved through confidence maps. By computing each pixel position(i, j) in the image $I^{n}$, the weighted canonical point map is computed by aggregating estimates from all relevant edges:

(1) $$\begin{array}{c} {\tilde{X}}_{n,i,j}=\frac{\sum_{e\epsilon {ℇ}^{n}}C_{n.e.i.j}X_{n,e,i,j}}{\sum_{e\epsilon {ℇ}^{n}}C_{n.e.i.j}}\text{,} \end{array}$$

where $X_{n,e,i,j}$ represents the obtained estimate value of $X_{n,n}$ from edge *e.* The canonical depth map is then extracted from the canonical point map ${\tilde{Z}}_{n}={\tilde{X}}_{n,:,:,3}$, and the Weiszfeld algorithm [37] is applied to optimize focal length:

(2) $$\begin{array}{c} f^{*}=\arg\min_{f}\sum_{i,j}{\left|\left|\left(i-\frac{W}{2},j-\frac{H}{2}\right)-f\frac{{\tilde{X}}_{n,i,j,1:2}}{{\tilde{X}}_{n,i,j,3}}\right|\right|}_{2}\text{,} \end{array}$$

assuming the pinhole model with central principal point and square pixels. To ensure the 3D point cloud strictly adheres to the pinhole camera model and precisely corresponds to pixel coordinates, a visibility-dependent constrained point map is constructed by defining camera extrinsics ${\;P}_{n}=\left[R_{n}/t_{n}\right]$ , intrinsics $K_{n}$, and scale factors $\sigma_{n}$, with the inverse projection formula for 3D points:

(3) $$\begin{array}{c} X_{n,i,j}=\frac{1}{\sigma_{n}}P_{n}^{-1}K_{n}^{-1}Z_{n,i,j}{\left[i,j,1\right]}^{⊺}\text{.} \end{array}$$

Based on the constructed constrained point maps and the result of camera parameter estimation, a hierarchical optimization strategy is employed to ensure global consistency. In this progress, we initially take advantage of pixel correspondences to achieve coarse alignment, and gradient descent is used to minimize a 3D matching loss:

(4) $$\begin{array}{c} \sigma^{*},P^{*}=\arg\min_{\sigma ,P}\sum_{\left(n,m\right)\epsilon E}\sum_{\left(i,j\right)\epsilon M_{n,m}}q_{c}{\left|\left|X_{c}^{n}-X_{c}^{m}\right|\right|}^{\lambda_{1}}\text{,} \end{array}$$

and this is performed iteratively using the Adam optimizer [38]. We reparameterize σ as $\sigma =\frac{\sigma^{\prime }}{\min\;\sigma }$ to ensure that the minimum value of σ is 1, which helps avoid degenerate solutions. Subsequently, the results from this coarse alignment undergo a second-stage global optimization. Local Bundle Adjustment (BA) is executed for each sub-scene to minimize a weighted 2D pixel reprojection error:

(5) $$\begin{array}{c} L_{2}=\sum_{\left(n,m\right)\epsilon E}\sum_{c\epsilon M_{n,m}}q_{c}\left[\rho \left(y_{c}^{n}-\pi_{n}\left({yX}_{c}^{m}\right)\right)+\rho \left(y_{c}^{m}-\pi_{m}\left({yX}_{c}^{n}\right)\right)\right]\text{,} \end{array}$$

which optimizes the camera extrinsic and intrinsic parameters along with the point cloud coordinates. After completing the above calculations, and in a manner similar to traditional SfM methods, anchor points are created and each pixel is rigidly connected to its nearest anchor point to form pseudo-trajectories. This process effectively reduces the number of optimization variables and enhances the model's optimization efficiency.

(2) Data format transformation

To utilize the output of the MASt3R model as the initialization object for 3DGS, it must be transformed into standard COLMAP-compatible SfM format: Extract valid 3D points from constraint point maps through merging and deduplication, then saved as points3D.txt, while storing camera intrinsic and extrinsic parameters following COLMAP specifications, where cameras.txt records focal length, principal point, and distortion coefficients; images.txt records the pose matrix and corresponding point observations for each image.

These formatted outputs can be directly integrated into the 3DGS workflow to achieve high-fidelity 3D reconstruction of plants.

### 2.4 High-fidelity rendering via 3DGS

To obtain high-fidelity 3D models of wheat plants, 3D Gaussian Splatting is used for reconstruction via rendering. The whole process is mainly divided into three stages: sparse point cloud initialization, Gaussian splat optimization, and visualization rendering.

#### 2.4.1 Sparse point cloud initialization

The initialization process of 3DGS is straightforward, beginning with sparse point clouds in SFM format reconstructed from multi-view images, serving as the initial positions of 3D Gaussian functions, each Gaussian point is represented by the formula:

(6) $$\begin{array}{c} G\left(x,\mu ,\sum \right)=\frac{1}{{\left(2\pi \right)}^{\frac{3}{2}}{\left|\sum \right|}^{\frac{1}{2}}}e^{-{\frac{1}{2}{\left(x-\mu \right)}^{T}\sum }^{-1}\left(x-\mu \right)}\text{,} \end{array}$$

where x is the position of any point in space, and μ represents the mean of the initialized Gaussian distribution, indicating the center position of each Gaussian point, $\sum_{3\times 3}$ is the covariance matrix, which determines the shape and orientation of the Gaussian distribution, parameterized by scaling factors s and quaternion q. Additionally, the opacity of the Gaussian splat is controlled by the parameter α, with a range of [0,1); Spherical Harmonic (SH) coefficients are used to control the color of the Gaussian distribution and can represent complex lighting effects. All parameters are updated during iterative optimization, and are rapidly splatted onto the rendered image through opacity blending projection.

To obtain high-fidelity 3D wheat model in the subsequent rendering process, the initialization stage begins from the positions of sparse points, using K-Means clustering to initialize Gaussian points' means μ based on the input sparse points. Assuming the input sparse point set $P=p_{1},p_{2}$*,* $p_{3}$ *…,* $p_{n}$,where $p_{i}$ is a point of the cloud, and $\mu_{i}$ represents the cluster's center, it is updated iteratively by the following formula:

(7) $$\begin{array}{c} \mu_{j}=\frac{1}{\left|C_{j}\right|}\sum_{p_{i}\epsilon C_{j}}p_{i}\text{,} \end{array}$$

where $C_{j}$ represents the set of all points in the j-th cluster, $\left|C_{j}\right|$ represents the number of points in the j-th cluster, and $\sum_{p_{i}\epsilon C_{j}}p_{i}$ represents the sum of position vector of all points in the cluster $C_{j}$.

The covariance matrix controls the scaling and rotation of the Gaussian splat, mathematically represented as

(8) $$\begin{array}{c} \sum_{3\times 3}=R\left(q\right)S\left(s\right){S\left(s\right)}^{T}{R\left(q\right)}^{T}\text{,} \end{array}$$

where R(q) represents the rotation transformation matrix q derived from quaternion, and S(s) represents the scaling transformation.

For the convenience of the model's learning, the initial covariance matrix is set to be isotropic, and the axis length of the Gaussian splat is equal to the average distance of the nearest three points, which ensures that the initialization size of the Gaussian function matches the geometric structure of the scene, avoiding excessively large or small initialization.

#### 2.4.2 Gaussian distribution optimization and visual rendering in plant reconstruction

The core of our approach is the optimization step, aiming to better fit the reconstructed model by adjusting the parameters of the Gaussian distribution, so that the rendered image is as consistent as possible with the input image. During the training process, each point in the space is expanded and projected onto a multi-view image for rendering. The quality of the training is judged by calculating the difference between the rendering result and the input image. We use Stochastic Gradient Descent to optimize the parameters of the Gaussian distribution, including position, covariance matrix, color and opacity. The loss function is $L_{1}$ combined with a D-SSIM term:

(9) $$\begin{array}{c} L=\left(1-\lambda \right)L_{1}+\lambda L_{D-SSIM}\text{,} \end{array}$$

and λ = 0.2 in our experiments, employing $L_{1}$ loss to ensure pixel-level precision in rendering processes, and D-SSIM loss to ensure the structural similarity of the rendering results. The contribution of both is balanced through weighting to achieve the parameter optimization objective.

After optimization warm-up, we conduct adaptive density control every 100 iterations, dynamically adjust the Gaussian distribution to optimize the fine-grained geometry of wheat organs, which ensures the integrity of wheat plant reconstruction. Gaussians are strategically added in high-detail regions, such as leaf structures, and reduced in areas of excessive reconstruction, with oversized Gaussians periodically removed from the spatial domain to optimize the balance between accuracy and computational efficiency.

In the rendering process, 3D Gaussians in the world coordinate system are projected to 2D rasterized plane in the camera coordinate system to enable effective interaction with camera parameters. This method defines the projected covariance matrix $\sum_{2\times 2}^{\prime }$ in camera coordinate as follows:

(10) $$\begin{array}{c} \sum_{2\times 2}^{\prime }=\text{JW}\sum W^{T}J^{T}\text{,} \end{array}$$

where J is the Jacobian matrix of the projection transformation and W is the view translation matrix relative to the initial camera pose $\left(R_{1},t_{1}\right)$, performing the transformation from the world coordinate system to the camera coordinate system.

A tile-based rasterizer approach is employed for rendering to achieve real-time rendering. Each pixel tile undergoes rasterization via 3D Gaussian projection transformation, instantiation, and global sorting operations. By adjusting the shape and orientations of Gaussians, anisotropic variance is rendered; the final pixel color is obtained through blending according to the opacity α of the Gaussians:

(11) $$\begin{array}{c} C=\sum_{i\epsilon N}c_{i}\alpha_{i}^{\prime }\prod_{j=1}^{i-1}\left(1-\alpha_{j}^{\prime }\right)\text{,} \end{array}$$

where $c_{i}$ is the color of each point and $\alpha_{i}^{\prime }$ is given by evaluating a 2D Gaussian with covariance **Σ** multiplied with a learned per-point opacity. Finally, we need to ensure the geometric positional relationship between the foreground objects and the background objects to guarantee physically plausible depth rendering.

### 2.5 Geometric computation based on Gaussian rendering

While 3DGS is excellent for high-fidelity rendering, the results usually need specialized renderers for visualization. Compared to that, point clouds, compatible with most 3D software, are extensively utilized in crop 3D phenotyping. Hence, this study employs the 3DGS-to-PC method [39] to quickly extract high-quality plant point clouds from the rendered 3D Gaussian scene, supporting further phenotypic analysis.

The process mainly includes regularization, color rendering, point sampling, and mesh generation, enabling robust extraction of high-quality point cloud representations from Gaussian scenes. First, a regularization term is introduced to process the Gaussian functions, ensuring that each covariance matrix is positive definite. Then, Gaussian filtering reduces the number of Gaussians in the scene by filtering large Gaussians and removing low-opacity Gaussians to produce accurate and plant-structure-consistent complex 3D Gaussians. In the color rendering step, considering the limitations of the traditional method that directly uses the Gaussian's own color and ignores the light change effect caused by the change of view angle, we simulate the rendering contribution of the Gaussian in the scene, calculate the color of each sampling point, use the Gaussian renderer for rendering, and perform reverse mapping according to the pixel color to ensure that the color is consistent with the real rendering result, improving the color authenticity and view-angle consistency of the plant point cloud. During the sampling process, use probability sampling to randomly sample from the multivariate normal distribution corresponding to the Gaussians, dynamically allocate the number of sampling points according to the volume of the Gaussian points to ensure that Gaussians with larger volumes can be allocated more points, and then use the Mahalanobis distance threshold to filter out abnormal points to ensure that the point cloud can accurately represent the plant structure. Subsequently, Open3D [40] was used to screen surface Gaussian points. Specifically, a statistical outlier removal filter(remove_statistical_outlier) was applied with optimized parameters (nb_neighbors and std_ratio) to eliminate isolated noise points generated by reflection and reconstruction artifacts. This parameter configuration was empirically optimized to balance noise removal and preservation of fine leaf details. The same processing pipeline was consistently applied to all samples across different growth stages to ensure data uniformity and geometric reliability. To prepare the point cloud for analysis, background elements (e.g., surrounding environment) were removed using an interactive cropping step. In this step, a 3D axis-aligned bounding box was first defined around each plant to roughly separate the plant canopy from the background. Points within the bounding box was retained, while points outside it were discarded. Subsequently, fine-grained manual editing was performed to remove residual background points adhering to the pot rim ensuring that only plant regions were retained for subsequent quantitative analysis.

For NeRF-based methods, since the geometry is represented implicitly as a density field, we utilized the Marching Cubes algorithm [41] to extract geometric structures. Specially, we evaluated the density field on a uniform voxel grid and extracted the isosurface with a predefined density threshold τ. The vertices of the extracted mesh were then treated as the representative point cloud for the subsequent quantitative comparisons against the point cloud generated by our Plant3R pipeline.

To evaluate the accuracy of the model - generated results, phenotypic parameters such as the plant height and volume (to verify the biomass) of the crops are extracted for verification. The height and volume of the plants are measured based on the point - cloud results and compared with the ground - truth measurements. To accurately compare the reconstruction results with the phenotypic parameters, the scale - recovery ratio of the model is established, using a standard checkerboard as a reference. After segmenting the plants, measure the plant height and volume. The plant height is determined by subtracting the z-coordinate of the pot edge from the z-coordinate of the highest point of the plant point cloud. The volume is calculated using the grid method, similar to integral calculus.

### 2.6 Implementation settings and model evaluate methods

All model training and evaluation experiments were conducted on Windows 10 operating system, using an Intel® Core™ i7-10700 CPU and an Nvidia GeForce RTX 4080 GPU. More information about key hardware components was shown in Table S2. Visual Studio Code was used as the programming environment, with Python 3.10 as the programming language. All other comparison algorithms were executed under this setting.

To quantitatively evaluate the rendering quality of our model, we employed several objective metrics to measure the differences between rendered and real images, including Peak Signal-to-Noise Ratio (PSNR), Structural Similarity Index Measure (SSIM), and Learned Perceptual Image Patch Similarity (LPIPS). PSNR represents the ratio between the maximum possible signal power of an image and the power of corrupting noise by calculating the mean square error (MSE) between the real and rendered images, and then converted to a logarithmic scale. As a crucial indicator of image quality, a higher PSNR value signifies better image quality. SSIM assesses the similarity between images by comprehensively comparing their luminance, contrast, and structural information; a value closer to 1 indicates higher structural similarity and better visual quality. LPIPS evaluates the perceptual similarity between images by extracting deep perceptual features using a pre-trained convolutional neural network. In this study, the AlexNet network [42] was selected as the feature extractor to derive features from rendered and actual images and evaluate their discrepancies. These metrics will collectively provide a comprehensive quantitative basis for our model's rendering performance.

## 3 Results

### 3.1 High-fidelity reconstruction efficiency

The Gaussian reconstruction of high-fidelity plants is divided into three parts, and the total time is approximately 50 min: among them, the acquisition of image data requires about 60 s; The time required for camera pose estimation and sparse point cloud reconstruction varies from 2 to 5 min depending on the complexity of the reconstructed scene. The initialization and rendering of plant point clouds are divided into 7000 training iterations and 30,000 training iterations. Among them, 7000 iterations take 3 to 5 min, and it takes approximately 20 min to complete 30,000 training iterations. After the 3D Gaussian rendering of the scene is completed, the time spent extracting the plant mesh depends on the structural complexity of the plants, basically ranging from 1 to 10 min.

Compared with the traditional COLMAP, which takes several hours to complete dense reconstruction and Poisson reconstruction to extract the accurate three-dimensional mesh of plants, Plant3R achieves faster convergence and higher-quality reconstruction results. This improvement mainly stems from the stronger initialization provided by the MASt3R-based sparse reconstruction, which allows the 3DGS stage to converge more efficiently without requiring additional views or longer optimization.

### 3.2 Accuracy analysis of camera pose estimation and initial point cloud extraction

The accuracy of camera pose estimation and initial point cloud directly impact the detail integrity of subsequent 3DGS rendering and the quality of dense reconstruction. To comprehensively and objectively evaluate the performance of the Plant3R framework against the traditional SfM algorithm, we conducted a quantitative assessment across four key wheat growth stages. The evaluation focuses on two dimensions: global statistic performance (Table 1) and plant-specific geometric fidelity (Table 2).

**Table 1:** Results of camera pose and sparse point cloud estimations.

	Method	Points	Observations	Mean Match Rate	Mean Observations Per Image	Mean Reprojection Error
Tillering	Plant3R	48028	180854	48.35%	6955.92	1.484
	SfM	10608	41638	34.05%	1604.46	1.118
Jointing	Plant3R	75770	263048	41.09%	8768.27	1.449
	SfM	18988	75217	36.43%	2507.23	1.143
Grain Filling	Plant3R	48037	171178	45.72%	6113.5	1.475
	SfM	9181	37934	32.37%	1354.79	1.116
Maturity	Plant3R	62890	216964	43.04%	7232.13	1.457
	SfM	11882	46816	31.84%	1560.53	1.137

Note: The term “Points” denote the number of points obtained in sparse reconstruction; “Observations” represent the total number of observations of all points across images; “Mean match rate” refers to the proportion of matched feature points among all observed points; “Mean observations per image” indicates the average number of observed points per image; “Mean reprojection error” describes the average reprojection error.

**Table 2:** Quantitative evaluation of sparse point cloud quality for different initialization methods.

	Method	Total Points	Plant Points	Plant Point Ratio	Canopy Coverage Index
Tillering	Plant3R	48028	3197	6.65%	12.1%
	SfM	10608	43	0.41%	0.3%
Jointing	Plant3R	75770	2684	3.54%	7.5%
	SfM	18988	117	0.62%	2.0%
Grain Filling	Plant3R	48037	5565	11.58%	14.9%
	SfM	9181	254	2.77%	0.1%
Maturity	Plant3R	62890	6237	9.92%	18.4%
	SfM	11882	461	3.88%	2.4%

Note: The term “Total Points” denote the number of points obtained in sparse reconstruction; “Plant Points” represent the number of the plant; “Plant Point Ratio” refers to the proportion of plant-region points to all reconstructed points $\left(\text{PPR}=N_{\text{plant}}/N_{\text{total}}\right)$; “Canopy Coverage Index” represents the proportion of occupied grid cells on the XY projection plane to the total grid cells within the plant's bounding box $\left(CCI=N_{\text{occ}}/N_{\text{total}}\right)$.

As presented in Table 1, the Plant3R model demonstrates a significant advantage in feature extraction and matching capabilities across the four key stages. The mean match rate of Plant3R consistently outperforms SfM, peaking at 48.35% during the Tillering stage – approximately 14% higher than SfM. Furthermore, the number of reconstructed sparse points generated by the Plant3R model was consistently several times greater than that of SfM. For instance, during the tillering stage, Plant3R reconstructed 48,028 points, whereas SfM produced only 10,608 points, and similar trends were maintained throughout the jointing, grain filling, and maturity stages. The mean observations per image for Plant3R are 3 to 5 times higher than those of SfM (e.g., 6995.92 vs 1604.46 at Tillering). This indicates that Plant3R establishes much richer connectivity between 2D images and 3D space. Regarding reprojection error, Plant3R has a higher mean reprojection error (∼1.45px) compared to SfM (∼1.12px). However, this apparent precision of SfM is achieved by aggressively filtering out feature points in low-texture regions, resulting in extremely sparse outputs. In contrast, Plant3R intentionally retains a larger number of challenging feature points, accepting a slight increase in pixel-level variance to preserve geometric completeness and ensure broader scene coverage.

To further analyze the geometric basis that is crucial for downstream 3DGS reconstruction is the plant rather than the background, an additional quantitative analysis focusing specifically on the plant region was performed (Table 2). While Table 1 establishes that Plant3R produces a denser and more feature-rich sparse reconstruction, Table 2 evaluates how much of this reconstruction geometry corresponds to the actual plant canopy. The results reveal that Plant3R consistently achieved a substantially higher number of valid plant points, plant point ratio (PPR), and canopy coverage index (CCI) than the SfM algorithm across all growth stages. For example, during the tillering stage, Plant3R produced 3197 plant points, accounting for 6.65% of all reconstructed points, while SfM yielded only 43 plant points (0.41%). At the maturity stage, Plant3R achieved 9.92% PPR and a canopy coverage index of 18.4%, whereas SfM achieved only 3.88% PPR and 2.4% CCI. The CCI, which quantifies the proportion of the canopy surface covered by the reconstructed points, clearly indicates that the Plant3R model not only generates more points overall but also captures a far greater proportion of points located on the biologically meaningful plant structures. This reflects the ability of Plant3R to recover more complete and continuous canopy geometry, especially in low-texture and geometrically complex regions that are challenging for traditional feature-based SfM methods.

In summary, the combined quantitative results in Table 1, Table 2 demonstrate that, during critical winter wheat growth stages, the Plant3R model significantly outperforms the traditional SfM algorithm in terms of feature extraction, matching, and the density of generated sparse point clouds. Although SfM holds a slight advantage in reprojection error, Plant3R's comprehensive point cloud generation capability is crucial for achieving high-quality dense reconstruction and detailed 3DGS rendering, which are key to accurate crop phenotyping analysis.

**Fig. 2:**
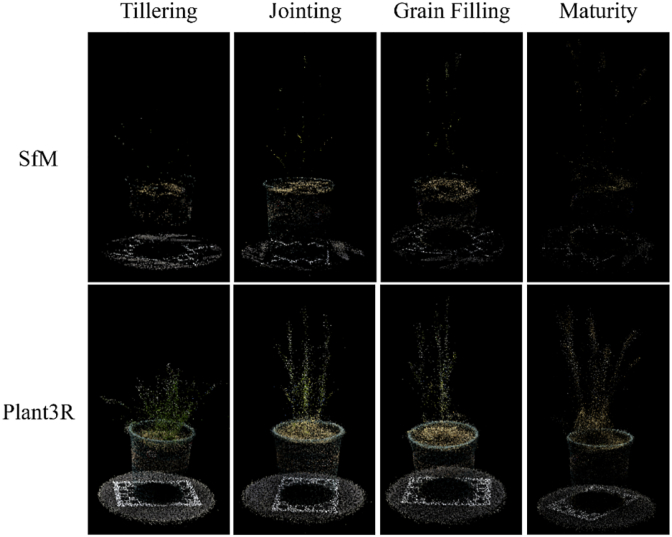
Comparison between SfM and Plant3R at different growth stages of wheat. In order to intuitively demonstrate the feature extraction and sparse point cloud reconstruction capabilities of the Plant3R model, we selected its reconstructed sparse point cloud for visual comparison with the SfM algorithm.

Fig. 2 illustrates the sparse point cloud visualization results for winter wheat potted plants obtained by both methods, directly confirming the quantitative differences mentioned above. As shown in Fig. 2, the point clouds reconstructed by the Plant3R model exhibit higher coverage and uniformity across the entire winter wheat canopy, especially in regions that are challenging for traditional methods, such as low-texture and complex geometric areas. In contrast, the point clouds generated by COLMAP appear sparse and uneven, with numerous significant holes in leaf and stem regions.

This difference in results stems fundamentally from the choice of image matching and feature extraction strategies. Compared to the conservative feature selection of traditional SfM algorithms, the Transformer-based architecture of the MASt3R model learns and infers matching relationships from a broader image context through point graph regression and dense correspondence. This enables the extraction of significantly more feature points, thereby substantially increasing point cloud density and the feature point matching rate, and demonstrating robustness and high coverage in complex scenes. This not only provides more accurate and richer geometric priors for the subsequent 3DGS model but also guarantees the stability of model convergence, particularly in low-texture plant reconstruction scenarios.

### 3.3 Gaussian rendering results of wheat

This section aims to evaluate the Plant3R model's real-time rendering and geometric mesh extraction performance using our experimentally collected wheat dataset. To quantitatively analyze the strengths and weaknesses of different algorithms, a multi-dimensional evaluation system was adopted. This system introduced metrics such as PSNR, SSIM, and LPIPS, in addition to visual comparisons. Compared to traditional SfM-MVS, NeRF, and 3DGS algorithms, this model exhibited more significant advantages in agricultural scene applications.

### 3.4 2D image rendering comparison

The following focus on thoroughly evaluating the 2D image rendering quality of the Plant3R model. We conducted a detailed analysis by comparing the rendering results of indoor wheat at different growth stages from the Plant3R model with those from Structure-from-Motion (SfM)-based NeRF and 3DGS algorithms.

**Fig. 3:**
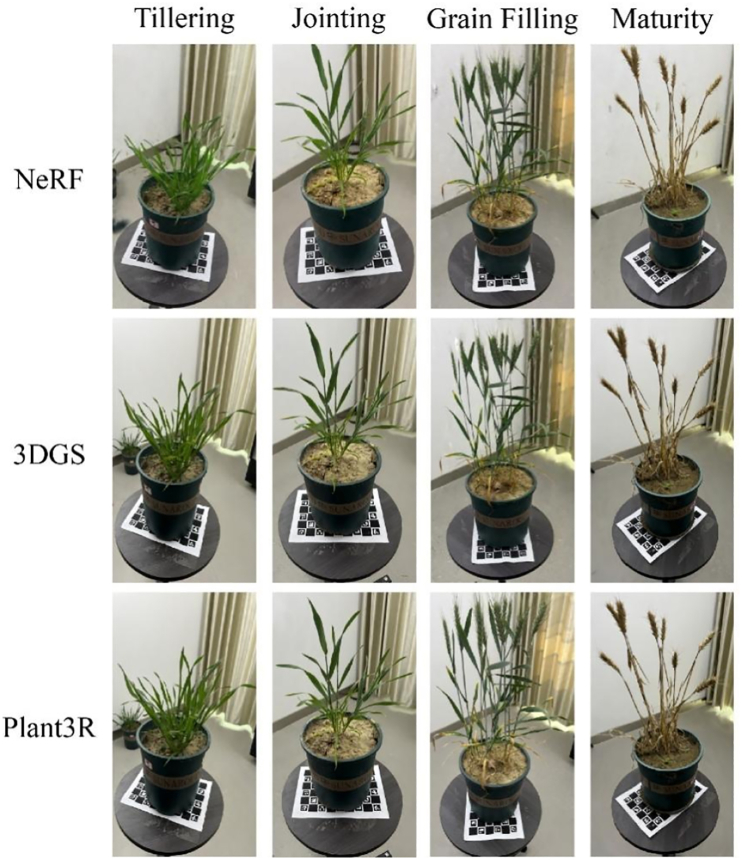
2D image rendering results of wheat's four growth stages under three different algorithms.

A comparison of the rendering results for NeRF, 3DGS, and Plant3R across different growth stages is presented in Fig. 3. From the visualizable results, our Plant3R model demonstrates significant advantages. Specifically, by integrating richer geometric prior information during the rendering initialization stage, Plant3R shows excellent results in handling artifacts and noise near the target object, effectively avoiding potential interference introduced by rendering noise. Furthermore, the Plant3R model is capable of capturing richer details with high fidelity when reconstructing wheat at different stages, including leaf textures, stem structures, and the microscopic morphology of wheat ears, with superior detail performance at high iteration counts. Although this model has exhibited superior rendering capabilities, we also note that it still inevitably shares some common issues inherent to 3DGS-based models. These limitations will be further discussed in subsequent sections.

From the comparison of metrics across different growth stages in Table 3, the Plant3R method generally demonstrates excellent rendering capabilities. Particularly in the tillering, jointing, and grain-filling stages, PSNR and SSIM reached their highest values, indicating that this method has a significant advantage in maintaining reconstructed geometric accuracy and texture details, enabling accurate reproduction of the plant's true morphology. Although the PSNR in the maturity stage was slightly lower than that of 3DGS, the difference was very minimal (e.g., the PSNR for the Plant3R model at the maturity stage was 30.62, while for 3DGS it was 30.98). Furthermore, the Plant3R model exhibited better overall stability, maintaining a high level of visual quality.

**Table 3:** Evaluation results of different 3D reconstruction methods for wheat at different growth stages.

	Metrics | Methods	NeRF	3DGS	Plant3R
Tillering	PSNR	27.44	32.61	34.03
	SSIM	0.89	0.92	0.92
	LIPIS	0.11	0.31	0.28
Jointing	PSNR	21.58	29.46	34.64
	SSIM	0.76	0.92	0.94
	LIPIS	0.27	0.30	0.29
Grain Filling	PSNR	26.18	29.64	34.02
	SSIM	0.87	0.91	0.94
	LIPIS	0.17	0.28	0.26
Maturity	PSNR	20.95	30.98	30.62
	SSIM	0.74	0.92	0.92
	LIPIS	0.27	0.28	0.27

The 3DGS method performed relatively consistently across all stages, particularly excelling in the LPIPS perceptual quality metric, showcasing its advantages in detail capture and realistic perception. This could be related to the sensitivity of its point cloud-based rendering mechanism to local details. In contrast, NeRF's metrics were significantly lower than the other two methods across all growth stages. This was especially noticeable in later growth stages where crop morphology is more complex, with a more pronounced decline in reconstruction accuracy and structural fidelity. This reflects the challenges NeRF may face when processing complex, non-rigid objects (such as plant leaves with fine textures and intricate structures), particularly when data volume or viewpoint coverage is insufficient.

Overall, the Plant3R method proposed in this study successfully combines the advantages of the MASt3R model in sparse point cloud generation with the characteristics of 3DGS in rendering efficiency and detail representation, thereby achieving higher quality rendering results. This method can more effectively preserve structural information in complex leaf textures and accurately reflect key agricultural phenotypic features, such as crop leaf edges and spikelet details at different growth stages, which is of great significance for crop 3D phenotyping research.

### 3.5 3D geometry extraction of wheat at different growth stages

**Fig. 4:**
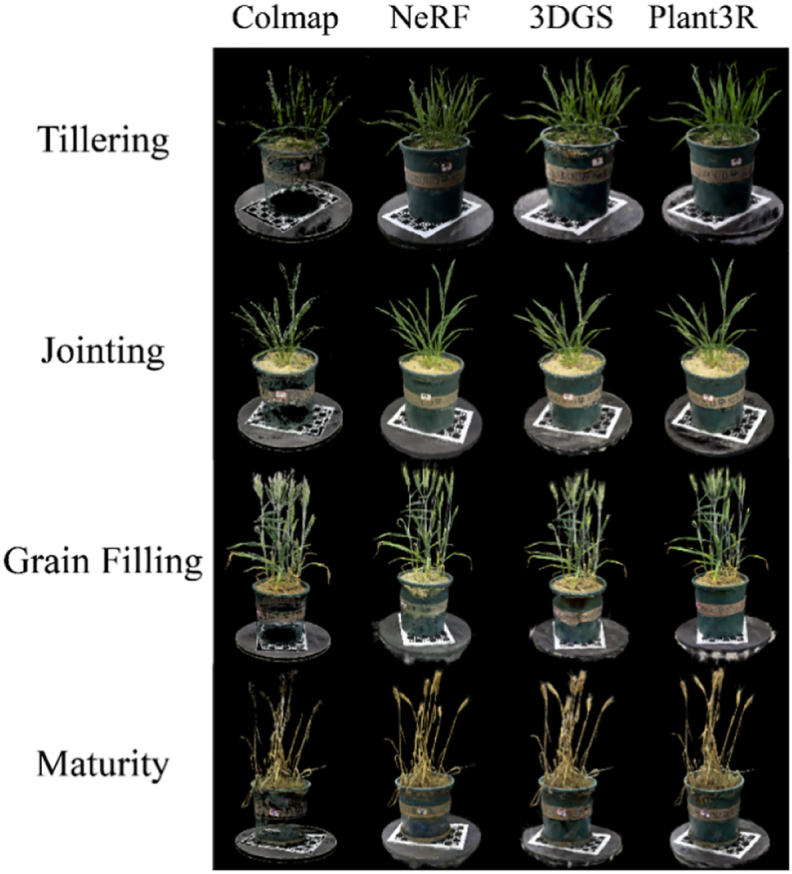
3D point cloud extraction results of wheat's four growth stages under four different algorithms. From the point cloud visualization results of different models, the fidelity of the Plant3R model is significantly better than Colmap, NeRF and original 3DGS.

High-quality 3D geometric results are crucial for accurate phenotypic analysis. They not only enable precise characterization of plant morphological features but also provide a structural basis for a deeper understanding of their physiological state. This is particularly important for the precise acquisition of information in complex and variable growth environments. To comprehensively evaluate our proposed Plant3R model, this section further compared its 3D geometric reconstruction capabilities with several widely recognized algorithms (Colmap, NeRF, and 3DGS). Fig. 4 visually presents the extracted geometric structures of wheat plants at different growth stages reconstructed by these various methods.

**Fig. 5:**
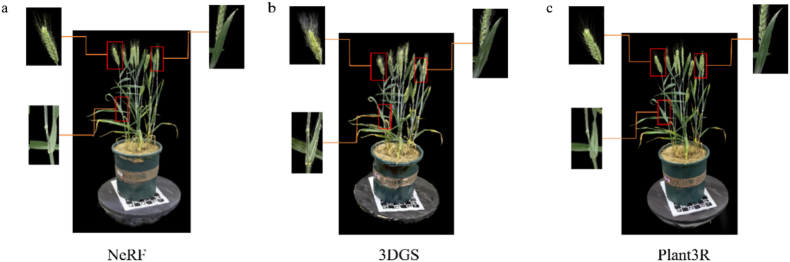
comparison between NeRF, 3DGS and Plant3R in model surface details such as leaf, stem and ear.

From the overall visual effect of Fig. 4, our Plant3R model demonstrates a clear advantage in 3D geometric reconstruction fidelity, structural completeness, and detail capture capability. The geometric results generated by Colmap exhibit significant incompleteness, particularly noticeable noise or omissions at the edges of plant leaves, failing to provide a smooth and continuous surface. In contrast, NeRF can achieve high-quality 2D image rendering, yet its geometric reconstruction results are sometimes accompanied by artifacts, surface blur, and insufficient representation of fine structures. The Plant3R model, however, is capable of reconstructing the complex 3D geometric morphology of wheat plants with higher precision and completeness. This includes clear and continuous stem forms, delicate spikelet structures, and even distinct texture details on the leaf surfaces, as shown in Fig. 5. This high-fidelity 3D reconstruction capability enables Plant3R to provide more robust and refined 3D wheat models for downstream plant phenotyping analysis across various growth contexts.

In summary, the Plant3R model's exceptional performance in 3D geometric reconstruction provides a solid data foundation for achieving high-precision, high-throughput wheat phenotyping analysis. This enhancement in capability is expected to improve efficiency and accuracy in the field of crop breeding and holds significant importance for advancing modern agricultural phenotyping research.

### 3.6 Crop phenotyping extraction and validation

To quantitatively verify the accuracy of crop 3D models in agricultural phenotyping, after completing the surface mesh extraction of wheat plants at different growth stages, this study further conducted a quantitative analysis of phenotypic parameters by comparing manually measured values of geometric phenotypic traits such as plant height, leaf length, and leaf width with model-calculated results. Considering the existence of a certain spatial scale relationship between the reconstructed plant models and actual plants, we used a standard 6x9 checkerboard paper as a calibration target to perform spatial scale recovery, ensuring the accuracy of subsequent phenotypic parameter extraction and its consistency with measured data.

**Fig. 6:**
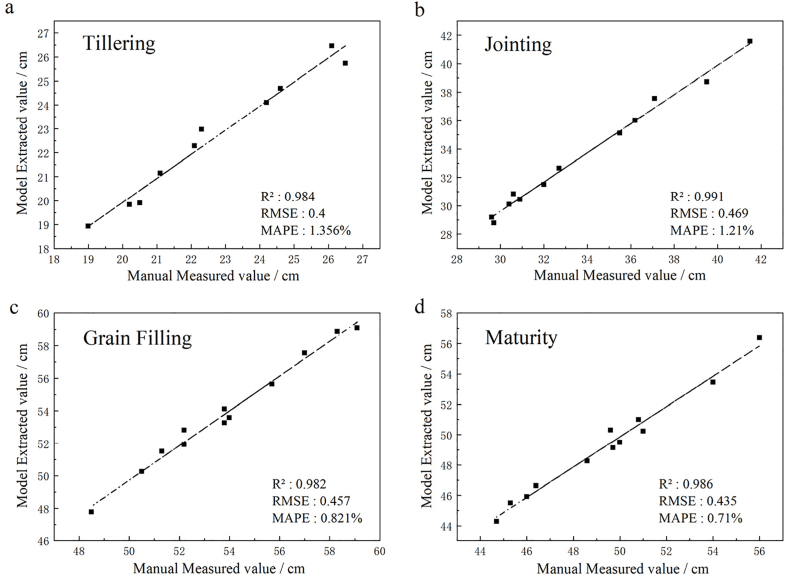
Comparison of model-derived and manually measured values of wheat plant height at different growth stages.

Plant height is an important indicator for evaluating the accuracy of reconstruction results. In this study, we defined plant height as the vertical distance between the soil plane and the highest point of the plant in the scale-recovered point cloud. To obtain a robust soil reference, the soil plane was estimated using RANSAC-based plane fitting from the lower region of the pot and soil points. The fitted plane was aligned to be parallel with OXZ plane to correct for potential tilt. The plant height(H) was then calculated as:

$$H=\max\left(y_{i}\right)-y_{plane}$$

where $\max\left(y_{i}\right)$ denotes the Y-coordinate of the highest point on the plant, and $y_{plane}$ represents the mean Y-coordinate of the fitted soil plane. The comparison with manual measurement data is shown in Fig. 6, and the results indicate that the R^2^ between the model-estimated plant height and the measured values across different growth stages is as high as 0.99. This demonstrates that the method proposed in this study can accurately extract wheat plant height information.

Due to the complex geometric structure of wheat plants—characterized by varying curvatures and mutual occlusions—extracting accurate phenotypic traits remains a challenge. To address this, we developed a Graph-based Geodesic Skeletonization algorithm to robustly quantify leaf morphology from the segmented 3D point clouds. First, to isolate target leaves from the reconstructed plant models, we performed interactive segmentation using CloudCompare software. For the segmented leaf point clouds, we constructed a Riemannian graph structure where nodes represent 3D points and edges connect nearest neighbors (k-NN) weighted by Euclidean distance. Leaf Length Definition: Unlike simple Euclidean distance which underestimates curved structures, we defined leaf length as the geodesic distance along the leaf's central topological skeleton. We utilized Dijkstra's algorithm to compute the shortest path within the graph from the petiole base to the leaf tip, followed by spline interpolation to generate a smooth, continuous central curve. Leaf Width Definition: Leaf width was calculated based on the local cross-sectional profile. For each node on the skeleton, we constructed a normal plane perpendicular to the tangent direction. The width was defined as the maximum span of the point cloud projection on this plane at the widest section of the leaf. This automated pipeline eliminates subjective errors associated with manual measurements and ensures robustness against leaf curling and twisting.

**Fig. 7:**
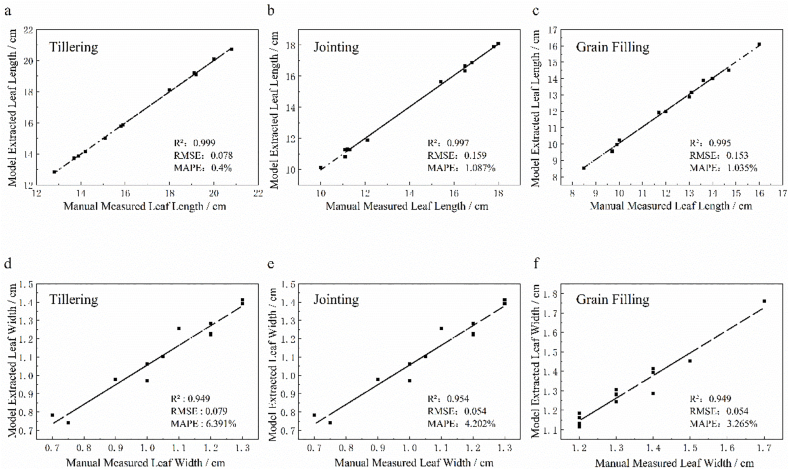
Validation of model-derived wheat leaf dimensions against manual measurements across different growth stages. Scatter plots showing the correlation between model-extracted and manually measured values for (a–c) leaf length and (d–f) leaf width across three growth stages (Tillering, Jointing, and Grain Filling). The high $R^{2}$ values (>0.94) and low RMSE indicate a strong agreement between the model estimations and ground truth.

To validate the accuracy of the proposed Plant3R reconstruction and the skeleton-based extraction method, we compared the model-extracted values with manual measurements (ground truth) obtained during the experiment. As shown in Table 4 and Fig. 7, the regression analysis demonstrates a strong correlation between the two methods across different growth stages (Tillering, Jointing, and Grain Filling). Specifically, the coefficient of determination (R^2^) for leaf length exceeded 0.99 with a Mean Absolute Percentage Error (MAPE) below 1.1%, indicating that the skeleton extraction algorithm effectively captures the true curvature of the leaves. For leaf width, the method also achieved high precision (R^2^ > 0.94, RMSE <0.16 cm).

**Table 4:** Comparison of model-extracted parameters and manually measured values of wheat leaf geometric indices at different growth stages.

		Scale Factor	Plant Height	Maximum Leaf Length	Maximum Leaf Width
Tillering	Extracted value	12.80	25.7	18.08	1.45
	Measured value	∖	25.6	18.22	1.40
Jointing	Extracted value	14.30	35.7	17.88	1.52
	Measured value	∖	35.2	18.03	1.50
Grain filling	Extracted value	13.85	50.9	12.04	1.61
	Measured value	∖	51.3	12.20	1.65
Maturity	Extracted value	14.75	49.4	∖	∖
	Measured value	∖	49.6	∖	∖

Note: The term “scale factor” is the conversion ratio that links the reconstructed point cloud's dimensions to the plant's true physical size, and it is directly influenced by the camera's focal length.

All the above validation results indicate that the wheat plants reconstructed based on the Plant3R model possess highly accurate geometric structures and effectively preserve the detailed features of the leaves. This provides reliable data support for subsequent precise plant phenotyping analysis based on 3D models.

## 4 Discussion

### 4.1 Plant3R's 3D reconstruction: enhancing accuracy through 3D feature learning and expanding agricultural applications

Existing 3D reconstruction methods, whether traditional MVS or the recently emerging NeRF and 3DGS, are highly dependent on the quality of SfM-based camera pose estimation and sparse reconstructed point clouds. They impose high demands on image resolution, overlap, and quality. When using COLMAP for data processing, reconstruction often fails or suffers from missing point clouds for certain key structures due to difficulties in feature matching. Our results clearly indicate that with the same data input, the Plant3R model achieved significant improvements in both feature point matching and sparse point cloud reconstruction. This enhancement is attributed to the Plant3R model's innovative approach of treating 2D image matching as a 3D task and establishing a cross-view 3D feature space through a cross-attention mechanism, thereby enhancing its ability to recognize low-texture and highly repetitive structures. Such robust and high-density initialization processing provides a more stable and richer data foundation for subsequent high-fidelity 3DGS rendering, effectively reducing the model's data requirements.

Building on this foundation, Plant3R fully leverages the precise pose estimation and dense reconstruction capabilities to achieve higher-fidelity plant 3D reconstruction and more accurate phenotypic feature extraction in conjunction with 3D Gaussian Splatting's rendering efficiency. Our experimental results show that the Plant3R model's metrics, such as PSNR and SSIM, surpass those of NeRF and the original 3DGS across key growth stages of wheat, including tillering, jointing, and grain-filling. It is capable of accurately capturing and restoring key phenotypic features like wheat leaf edges and spikes. This high-fidelity reconstruction not only provides a solid foundation for accurate phenotypic parameter extraction but also offers high-resolution 3D data support for a deeper exploration of genotype-phenotype-environment interaction relationships.

### 4.2 Limitations and future potential

Despite the high reconstruction accuracy and robustness demonstrated by Plant3R model across different growth stages of wheat, several limitations warrant further investigation and provide clear directions for future research.

1) Adaptability to more complex environments

This study focused primarily on the reconstruction of potted wheat plants, where environmental control is relatively ideal. When the model is applied in open-field environments, its reconstruction accuracy and robustness may be affected by factors such as complex lighting changes, wind-induced plant motion, and background occlusion. Future research can explore how to integrate 3D reconstruction techniques for dynamic scenes (e.g., methods based on event cameras or dynamic Gaussian fields) with the Plant3R model to adapt to more challenging field environments.

2) Multimodal data fusion and deep phenotypic analysis

Currently, the majority of research relies on RGB images for 3D reconstruction. However, certain physiological or pathological information of wheat plants (e.g., nitrogen content, water stress) cannot be acquired solely through RGB images. Future work can consider integrating multimodal data, such as by utilizing multispectral or hyperspectral imaging technology, to fuse spectral information with 3D structural information. This would enable a more comprehensive and in-depth analysis of plant phenotypic features, providing richer data support for precision agriculture and crop stress diagnosis.

3) Applicability and generalization across plant scales and species

The current version of Plant3R has been validated mainly on wheat plants ranging from approximately 20-60 cm, covering different key growth stages where the topology and geometry vary greatly and are similar to other cereal species (e.g., rice, barely and oat). These developmental differences already test the model's robustness to major morphological changes within one species. While additional plant species were not included in this study, the data- and species-agnostic design of Plant3R -reconstructing plant geometry from plant 3D geometric priors rather than crop-specific parameters-suggests good transferability to crops with comparable morphology.

For taller and more complex crops like maize or sorghum, the framework is also adaptable, even though the additional vertical layers and self-occlusion pose present challenges for image coverage. To address this, we recommend a “multi-tier cylindrical” acquisition strategy: (1) Stratified Sampling: capturing images at multiple elevation levels to ensure uniform point density. (2) Upward Views: specifically adding upward-looking camera angles at the bottom tier to capture the stem base and the underside of leaves, which are often occluded in standard top-down views. This adaptive approach support scalable application of Plant3R across diverse crop architectures.

## 5 Conclusion

This study successfully proposed and validated an innovative 3D reconstruction method for potted wheat plants: the Plant3R model. This model effectively combines the MASt3R model's advantage of providing stable and accurate geometric priors with 3D Gaussian Splatting's high-fidelity rendering capability. Our results demonstrated that the Plant3R model significantly outperforms the traditional SfM algorithm in terms of feature extraction, matching, and the density of generated sparse point clouds. This effectively enhances reconstruction accuracy and robustness when dealing with complex, low-texture, and highly repetitive feature scenes like wheat plants. In the rendering results evaluation, the Plant3R model's performance surpassed other methods, including the original 3DGS, achieving a PSNR over 34 and an SSIM of 0.94. The average relative error between phenotypes extracted from the reconstructed 3D models and manual measurement results was within 6% which fully verifies the quantitative analysis accuracy of this method in the 3D model accuracy validation. Consequently, the Plant3R model offers significant practical utility, which is reflected not only in its ability to improve the fidelity of plant reconstruction but also in providing a new technical pathway and methodological reference for subsequent high-throughput phenotyping research. Moving forward, with the development of automated imaging systems and multimodal data acquisition methods, the Plant3R model holds promise for further expanding its application potential in crop breeding, smart agriculture, and precision management.

## Author contributions

X.H. proposed conceptualization. J.M. conducted the methodology. J.M. and Y.Z. wrote the original manuscript. J.M., Y.Z., X.H., and L.S. revised the manuscript. L.S., X.H. and Y.Z. applied for funding. L.S. and X.H. provided experiment resources. J.M. and Y.J. performed visualization. J.M., Y.J., H.Z., and S.D. acquired the data under Y.Z.’s supervision. J.M. and Y.J. conducted the data analysis.

## Funding

This work was supported by the National Natural Science Foundation of China (No. 52425901 and No. 52309058).

## Declaration of competing interest

The authors declare that they have no known competing financial interests or personal relationships that could have appeared to influence the work reported in this paper.

## Data Availability

The data that support this study are available upon reasonable request from the corresponding author. Code is available at Mlynnray/Plant3R.
