# Supplementary material for: Plant3R: Fusing 3D feature learning with Gaussian splatting to enhance wheat plant 3D reconstruction precision
Source: Plant Phenomics. 2026 Mar 21;8(2):100200. doi: 10.1016/j.plaphe.2026.100200 (PMC13316467; doi:10.1016/j.plaphe.2026.100200)
Supplement: Multimedia component 1 [file mmc1.docx]

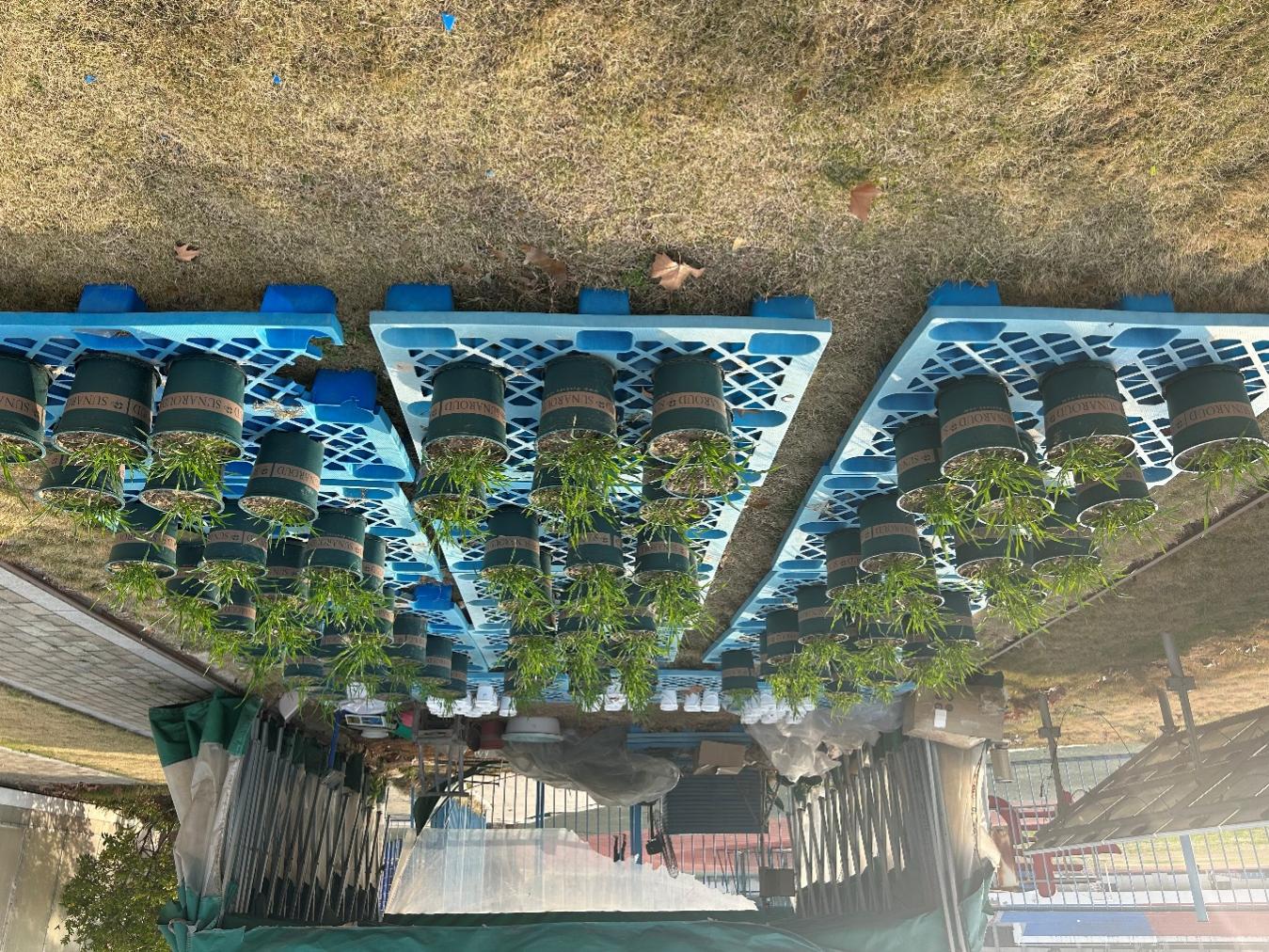


**Fig. S1.**  A schematic illustration of the initial placement positions of potted plants in the experimental field. All potted plants were arranged in accordance with the pre - designed layout. This was done to ensure a uniform spacing among various experimental units and enable their growth under identical environmental conditions.

Table S1 – Detailed specifications of the imaging device, acquisition settings, and dataset composition.

|  | Parameter | Value/Description |
| --- | --- | --- |
| Imaging Device | Model | iPhone 14 (Main Camera) |
|  | Sensor Resolution | 4032×3024 pixels |
|  | Aperture | f/1.5 |
|  | Focal Length | 26 mm (35 mm equivalent) |
| Acquisition Settings | Trajectory | Hemispherical (360° around the plant) |
|  | Image Overlap | Approx. 75%–80% between adjacent views |
|  | Images per Plant | 30 (Fixed for all samples) |
| Dataset Composition | Growth Stages | Tillering, Jointing, Grain Filling, Maturity (4 stages) |
|  | Samples per Stage | 12 independent plants |
|  | Total Scenes | 48 scenes (4 stages × 12 plants) |

Table S2 – the information of key hardware components used for data processing in this study.

| Hardware component | Model |
| --- | --- |
| Motherboard | AUROS B460M |
| GPU | NVIDIA GeForce RTX 4080 (16 GB) |
| CPU | Intel (R) Core (TM)i7-10700 CPU @ 2.90GHz |
| RAM | 32 GB |
